# Land use change influences soil C, N, and P stoichiometry under ‘Grain-to-Green Program’ in China

**DOI:** 10.1038/srep10195

**Published:** 2015-05-19

**Authors:** Zhao Fazhu, Sun Jiao, Ren Chengjie, Kang Di, Deng Jian, Han Xinhui, Yang Gaihe, Feng Yongzhong, Ren Guangxin

**Affiliations:** 1 College of Agronomy, Northwest A&F University, Yangling, 712100 Shaanxi, China; 2 The Research Center of Recycle Agricultural Engineering and Technology of Shaanxi Province, Yangling 712100 Shaanxi, China; 3 College of Forestry, Northwest A&F University, Yangling, 712100 Shaanxi, China; 4 College of Life Science, Northwest A&F University, Yangling, 712100 Shaanxi, China

## Abstract

Changes in land use might affect the combined C, N and P stoichiometry in soil. The Grain-to-Green Program (GTGP), which converts low-yield croplands or abandoned lands into forest, shrub, and/or grassland, was the largest land reforestation project in China. This study collected the reported C, N and P contents of soil in GTGP zones to achieve the factors driving the changes in the C:N, C:P, and N:P values. The results showed that the annual average precipitation exerted significant effects on the C:P value, and on the N:P value became significant 20 years after the change in land use. The annual average temperature was the main factor affecting the C:N value during the first 10 years, while the annual average precipitation strongly affected this value afterwards. In addition, “Redfield-like” interactions between C, N, and P in the soil may exist. A linear regression revealed significant positive correlations between the C:N, C:P, and N:P values and the restoration age, temperature, and precipitation after a change in land use. Therefore large-scale changes in land use under the ‘GTGP’ program might significantly affect the C:N, C:P and N:P ratios in soil.

Elements and their ratios in environments affect organisms, while organisms could also affect the elemental compositions in their environments by absorbing or releasing different elements^1^. Carbon (C), nitrogen (N) and phosphorus (P) are the three major elements in living organisms; these elements occur in relatively stable ratios. The key characteristics of organisms and ecosystems are determined by the dynamics of the element ratios^2^,^3^. Redfield reported that planktonic biomass contains C, N, and P in a comparatively steady atomic ratio of 106:16:1, similar to the proportions of C, N, and P in marine water, and this chemical relationship was named the “Redfield ratio”^4^. Although investigations of the ‘Redfield ratio’ have generated a deep understanding of the biological processes occurring in ocean ecosystems, the C, N, and P stoichiometries in terrestrial ecosystems remain poorly understood^5^,^6^.

Unlike marine ecosystems, which are characterized by strong wave-mixing, terrestrial ecosystems invariably exhibit significant spatial heterogeneity among the biogenic elements (i.e., C, N and P) due to long-term ecological differences (i.e., geology, topography, and climate) and short-term ecological disturbances (i.e., natural disaster, anthropogenic)^7^. In recent decades, great progress regarding the C, N, and P stoichiometry has been made in terrestrial ecosystems, such as plants leaves and litter^5^,^8^,^9^, forests^10^, and microorganisms^11^,^12^. In response to nutrient supplies in plants, the C, N, and P stoichiometry in the soil is critical in terrestrial ecosystems and far more complex than in other terrestrial systems.

Previous studies indicated that the organic C and P in soil are well-constrained based on an analysis of 22 grassland soils^13^. Strong correlations between the total C and N in the soil were also found^14^,^15^. Moreover, a recent study reported that the C:N ratio in soil is a useful indicator of the decomposition of organic soil matter^16^,^17^, while the N:P ratio also has potential diagnostic value because it is changed by fertilization^18^. Additionally, Tian *et al.* reported that the P supply in soil depends on the total P content and the weathering stage of the parent material, which is characterized by spatial heterogeneities^19^. In addition, this work proposed a ‘Redfield ratio’ in soil^19^. Cleveland and Liptzin^12^ reported that the C, N, and P stoichiometry in soil remains relatively stable at 186:13:1 on the global scale. Lal^20^ suggested that the humus C:N:P:S ratio is 10,000:833:200:143. Tian *et al.*^19^ found a well-constrained C:N molar ratio (14.4), as well as relatively consistent C:P (136) and N:P (9.3) ratios, with a general C:N:P ratio of 134:9:1. Cristina *et al.*^21^ also demonstrated that the interactions among the season, vegetation type and structure, and soil properties affect microbial nutrient immobilization under a Mediterranean-type climate to influence the biogeochemical cycles for C, N and P in Mediterranean forest ecosystems. To date, studies on the soil C, N, and P stoichiometry at different scales are lacking, and information about their influences on the global or regional scale are scarce, particularly in China.

In China, widespread ecological degradation has constrained sustainable socioeconomic development in recent decades, particularly before the end of the 20th century^22^. After the 1950s, the Chinese government has made great efforts to control soil erosion and restore ecosystems^23^. More than 9.27 million ha of cropland and abandoned land have been afforested in China through the “Grain to Green Program” (GTGP), which has required more than 28.8 billion USD and involved 0.12 billion farmers; the GTGP has implemented large-scale ecological rehabilitation since 1999^22^. Currently, this is the first and most ambitious “payment–for–ecosystem–services” program in China^22^,^24^. Although the initial goal of the GTGP was to control soil erosion, the program strongly affects the C, N, and P cycling in soil^25^,^26^. However, few studies have reported the soil C, N, and P stoichiometries under GTGP. Therefore, this study aims to accomplish the following: a) illustrate the distribution of the soil C:N, C:P, N:P values under the GTGP; b) establish the changes in the soil C:N, C:P, N:P values after the change in land use; and c) study the factors driving the changes in the C:N, C:P, N:P ratios.

## Results

### Changes in the soil C:N, C:P, and N:P values due to the ‘Grain-to-Green Program’

**Figure 1:**
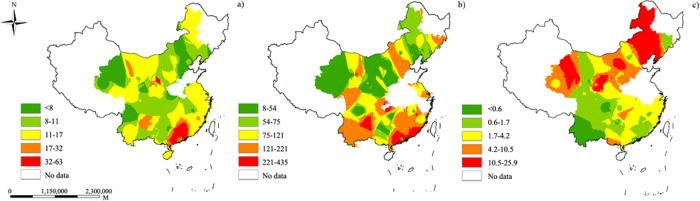
Distribution of soil C:N value (**a**), C:P value (**b**) and N:P value (**c**) value under ‘Grain-to-Green Program related zones’. The map plotted by Arcgis9.3 using inverse distance weighting (IDW) method.

**Figure 2:**
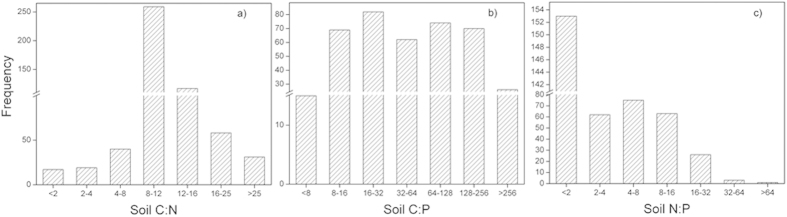
Frequency distribution of the soil C:N (**a**), C:P (**b**) and N:P ratios (**c**) in the China ‘Grain-to-Green Program’.

The distribution of the soil C:N, C:P, and N:P values from 0–20 cm measured after the change in land use under the ‘Grain-to-Green Program’ Program in China is displayed in Fig. 1. The highest soil C:N and C:P values were obtained in Southern China (Guangdong and Guangxi province) (Fig 1a,b), while the highest soil N:P values were obtained in Northern China (Jiling and Heilongjiang province) (Fig 1c). The frequency distribution of the soil C:N, C:P, and N:P values (Fig. 2) revealed that most of the soil C:N, C:P, and N:P values were ranged from 8 to 16, 16 to 32, 0 to 2, respectively.

### Changes in the soil C:N, C:P, and N:P ratios in response to the changes in land use

**Figure 3:**
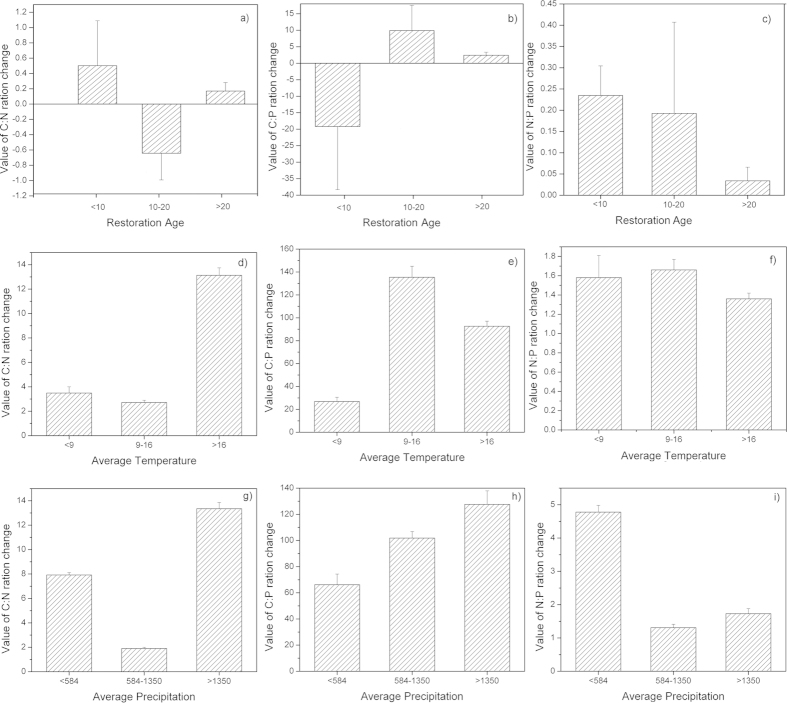
Change in the soil C:N (**a**), C:P (**b**), and N:P (**c**) ratios as a function of the restoration age after the change of slop cropland or abandoned land to forest. The error bars represent the standard errors for the slope of Equation 3 (k), and the values above the bars are the corresponding number of observations (the meaning of the error bars and the values are the same in Figs. 5,6).

*For forest* The effects of the change in land use from cropland or abandon land to forest on the soil C:N, C:P, and N:P values changes are shown in Fig. 3a–c. The changes of soil C:N ratio increased during the first 10 years and after 20 years (Fig. 3a) but decreased from 10 to 20 years. In contrast, the changes of soil C:P ration decreased over the first 10 years before increasing (Fig. 3b). The changes of soil N:P ratio constantly increased after the change in land use; the highest changes of ratios were achieved during the first 10 years. Meanwhile, the changes of soil C:N, C:P, and N:P ratios were differently following annual average temperature (Fig. 3d–f). The changes of soil C:N ratio was highest in >16 °C, whereas the changes of C:P, and N:P ratio were highest in 9–16 °C. However, the changes of soil C:N, and C:P ratio were highest in >1350 mm following annual average precipitation, while the changes in the soil N:P ratio was found in <584 mm (Fig. 3g–i).

**Figure 4:**
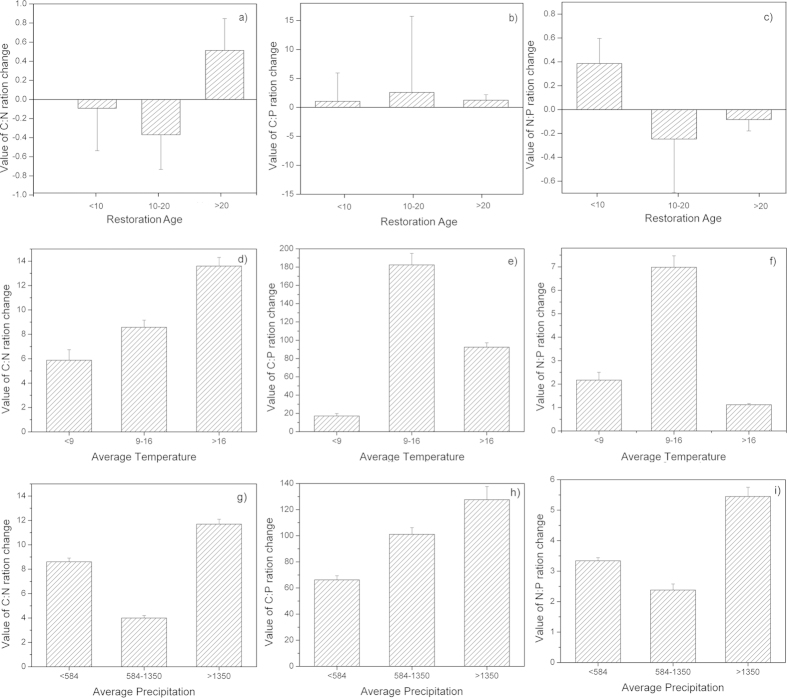
Change in the soil C:N (**a**), C:P (**b**), and N:P (**c**) ratios as a function of the restoration age under the ‘Grain-to-Green Program’ for converted shrublands.

*For shrubland* generated by a change in the land used for cropland or abandoned land, the changes of soil C:N ratio decreased slightly during the first 10 years and decreased sharply during years 10 to 20 before increasing after 20 years (Fig. 4a). The changes in the soil C:P ratio increased continuously after the changes in land use; the highest changes of ratios were obtained from years 10 to 20 (Fig. 4b). Additionally, the changes of soil N:P ratios increased during the first 10 years before decreasing continuously (Fig. 4c). The changes in the soil C:N, C:P, and N:P ratios were different with annual average temperature (Fig. 4d–f). the changes in the soil C:P, and N:P ratio were highest in 9–16 °C, but the changes in C:N ratio was highest in >16 °C. Moreover, the changes in the soil C:N, C:P, and N:P ratios were all highest in >1350 mm following average precipitation (Fig. 4g–i).

**Figure 5:**
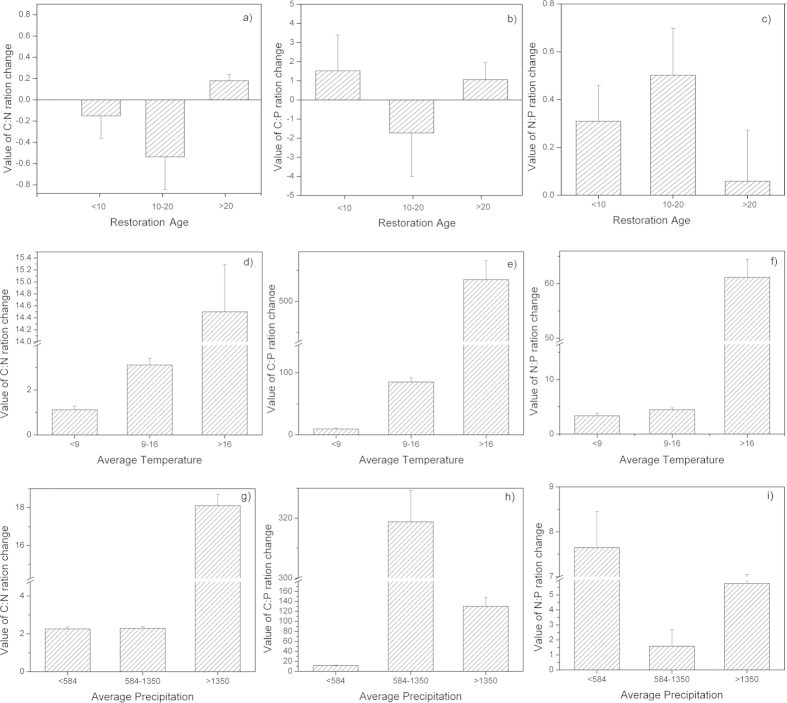
Change in the soil C:N (**a**), C:P (**b**), and N:P (**c**) ratios as a function of the restoration age under the ‘Grain-to-Green Program’ for converted grasslands.

*For grassland* generated from cropland or abandoned land, the changes in the soil C:N ratio decreased slightly during the first 10 years before decreasing sharply during 10 to 20 years and increasing after 20 years, similarly to shrubland (Fig. 5a). Meanwhile, the changes in the soil C:P ratio increased during the first 10 years and after 20 years but decreased during 10 to 20 years (Fig. 5b). However, the changes in the soil N:P ratio increased during all stages after the changes in land use, and the highest value appeared during years 10 to 20 (Fig. 5c). Meanwhile, The changes of soil C:N, C:P, and N:P ratios were highest in >16 °C following annual average temperature (Fig. 5d–f), however, there was no obvious regularity following average precipitation (Fig. 5g–i).

### Factors affecting the soil C:N, C:P, and N:P values

Stepwise regressions revealed that the annual average temperature was the main factor affecting the soil C:N value during the first 10 years, and the annual average precipitation had significant effects on the soil C:N value after 10 years (Table. 1). The annual average precipitation also significantly affected the soil C:P value continuously after the change in land use. The restoration age was the major factor affecting the soil N:P value during the first 20 years, whereas the annual average precipitation strongly affected the soil N:P value 20 years after the change in land use. ANOVA shown that significant positive correlations between the soil C:N, C:P, and N:P values and the restoration age, temperature, and precipitation after the change in land use (p < 0.05), but their interactions were significant in few of case (Table. 2).

## Discussion

The soil C:N, C:P, and N:P ratios are good indicators of the status of soil nutrients during development^19^. the high C:N ratios (>25 on a mass basis) indicate that organic matter is accumulating faster than it is decomposing. Our results showed that most of the soil C:N value ranged from 8 to 16 (Fig. 2), indicating that the organic matter is thoroughly broken down. Similar results were reported by Bui and Henderson^27^. Our synthesis also revealed that most of the soil C:P value ranged from 16 to 32 (Fig. 2), implying a net mineralization of nutrients. Paul^16^ also reported that C:P <200 implies a net mineralization, C:P >300 implies a net immobilization, and a C:P between 200 and 300 reveals little change in the soluble P concentrations.

Cleveland and Liptzin^12^ estimated that the global soil C:P and N:P ratios for surface soil (0–10 cm) are 186 and 13.1, respectively. Tian *et al.*^19^ reported that the C:P and N:P values were 136 and 9.3 at the same depth in China. Our analysis revealed lower values. These differences might occur because the soil samples used by Cleveland and Liptzin^12^ and Tian *et al.*^19^ have a humified litter layer, generating higher C:N, C:P, and N:P values. Additionally, the correlation between the total soil C, N and P was obtained based on more than 592 soil samples (Table. 3). The results revealed that the C:N ratio was highly constrained based on the relatively high correlation coefficient (0.71) for the C and N concentrations. Relatively constrained C:P and N:P ratios were observed with correlation coefficients of 0.28 and 0.48, respectively, implying a relatively constrained C:N:P ratio, similarly to that reported by Cleveland and Liptzin^12^ and Tian *et al.*^19^. Therefore, we agree with Cleveland and Liptzin^12^ that “Redfield-like” interactions may exist among C, N, and P in soil.

The C, N, and P stoichiometry in soil varies based on the type of land use, and these variations are highly complex^8^. Our results indicated that the effects on the soil C:N, C:P, and N:P values in the forest, shrub and grassland varied (Figs. 3,4,5). For example, the conversion of cropland or abandoned land to forest increased the C:N value over the first 10 years (Fig. 3a), these values decreased during the same period in land that was converted into shrub and grassland (Figs. 4a,5a). These differences most likely occur for two reasons. Plants change the C, N, and P ratios by absorbing/releasing these elements from/to soil^28^,^29^. However, soils with different vegetation undergo different litter decomposition processes and rates, meaning that the release of nitrogen and phosphorus to soil differs^6^. Similar results were reported by Elisabeth and Brent^27^. However, our results contrasted sharply with those of Cleveland and Liptzin^12^, who reported that the soil nutrient ratios did not vary significantly between forests and grasslands. We speculate that vegetation covers, plant communities, and geomorphology all affect the nutrient stoichiometry in soil. Li *et al.*^7^ reported that the different types of land use exhibited different soil C:N:P ratios due to differences in elevation, vegetation type and land management practices. Aponte *et al.*^30^ presented a Spanish dataset indicating that the average soil C:N:P ratio in forests is slightly greater than that in woodlands; this ratio varied based on the type of land use. Additionally, compared to other countries, the soil C:N, C:P, and N:P values in China also vary between forest, shrub and grassland (Table. 4). For example, the soil C:N, C:P, and N:P values in China were lower than in some countries (i.e., USA, Germany) and were higher than those found in other countries (i.e., UK). These variations likely arose from the different climatic zones, soil orders, soil depth and weathering stages, which affect the soil C:N, C:P, and N:P values. Similar results were reported by Tian *et al.*^19^and Zhang *et al.*^31^.

The restoration age, temperature and precipitation are important factors that must be considered when estimating the soil C, N, and P stoichiometry after changing the land use (Table. 1, Figs. 3,4,5, and Table. 2). A stepwise regression revealed that the annual average temperature was the primary factor affecting the soil C:N value over the first 10 years. However, the restoration age became the major factor affecting the soil N:P value during first 20 years (Table. 1). In addition, the annual average precipitation also significantly affected the soil C:P value during all of the stages after the land use change (Table. 1). Therefore, the C, N, and P ecological stoichiometry is highly complex in soils. The climate may affect the soil C, N, and P stoichiometry that accumulates through biotic processes, relying on the productivity of the vegetation and the decomposition of organic matter. Several studies have illustrated that the climate imposes important controls on the biota and its interaction with the soil nutrients^15^,^30^,^31^,^32^. Moreover, the restoration of the soil C content after afforestation varies with the climate^26^,^33^,^34^,^35^,^36^. The annual average temperature and precipitation also affected the soil organic carbon during some stages after the change in land use^27^. In addition, Zhang *et al.*^30^ reported that the soil C:N value was primarily affected by the total phosphorus, the soil C:P value was primarily affected by the total nitrogen, and the soil N:P value was primarily affected by the soil organic carbon. Therefore, the C, N, and P stoichiometry in soils is also affected by the soil elements, which are largely influenced by the annual average temperature and precipitation.

## Methods

**Figure 6:**
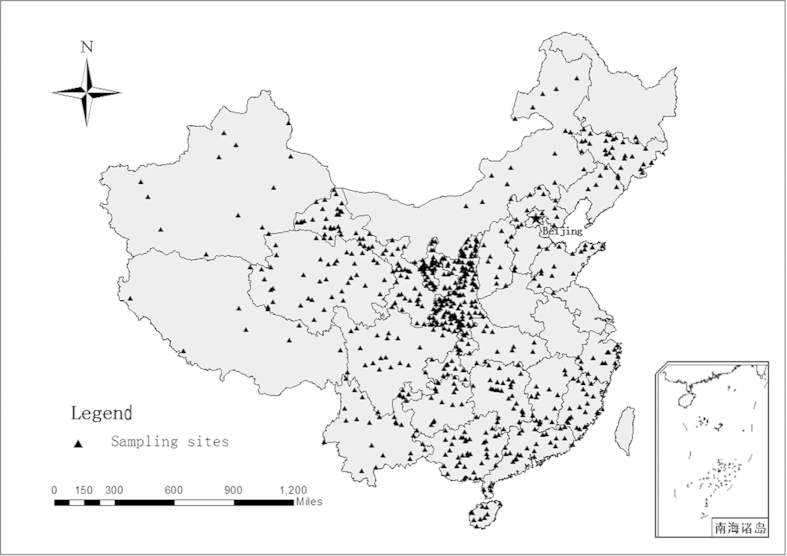
Distribution of sampling sites in the dataset. The map plotted by Arcgis9.3.

All of the available publications concerning the changes in the C, N, and P contents in soil from forest, shrub, and grassland that was converted from cropland and/or abandoned land from 1999–2013 under the GTGP in China were collected. The following criteria were used to select publications for analysis:

- data existed for the all land use types (grasslands and shrublands as well as forest) sites;
- The soil C, N, which were measured by the K_2_Cr_2_O_7_-H_2_SO_4_ oxidation method and the Kjeldahl digestion procedure method, the phosphorus method was used complexation with ammonium molybdate and antimony potassium tartrate followed by quantification using a spectrophotometer. The contents of at least two of the elements (C, N, and P) were provided or could be calculated;
- paired sites were used within their chronosequence (sampled many replicate plots and paired plots over a landscape, those plots with the same age, edaphic conditions and land use were pooled);
- similar conditions (i.e., soil types, elevation);
- the restoration age (year), temperature (°C), and precipitation (mm) were clearly given;
- additionally, studies that lack replication or provide unclear information were excluded;
- The reported sites were distributed across the GTGP zones shown in Fig. 6.
- The final dataset was composed of 92 publications that included 592 observations (Appendix 1).

In our study, the C:N, C:P, and N:P ratios are calculated on a molar basis. The raw data were either obtained from tables or extracted by digitizing graphs with the GetData Graph Digitizer (version 2.24, Russian Federation) as reported by Deng *et al.*^37^. The following information was compiled from each publication: location (longitude and latitude), annual temperature and precipitation, types of land use (forest, shrub, or grassland), and restoration age after the change in land use. The soil layer was set to 0–20 cm because most works only documented the changes in the C, N, and P contents within this layer, and significant differences were only observed in the topsoil^23^. Moreover, Tian *et al.*^19^ reported that the soil C:N, C:P, and N:P ratios in organic-rich topsoil might be a good indicator of the soil nutrient status during soil development. Studies utilizing different soil depths (for example, 5 publications utilized 27 observations at 0 – 15 cm; and 7 publications utilized 49 observations at 0 – 10 cm and 10 – 20 cm) were adjusted to encompass 0~20 cm. In this study, we assumed that no differences were observed in the C, N, and P contents from 15 to 2  cm. Specifically, the changes in the C, N, and P contents in the 0~15 cm layer equaled those in the 0~20 cm layer. Averaged values from 0 – 10 cm and 10 – 20 cm were used to represent the 0 – 20 cm layer. The restoration age of afforestation was divided into three groups: <10, 10 – 20, and >20 years. The previous type of land use was cropland or abandoned land in all cases.

If a sample only reports the SOM, the SOC value is calculated using the following formula^38^:


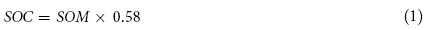


where SOC is the soil organic carbon and SOM is the soil organic matterIf the bulk density (BD) of the soil is not reported, it is calculated^28^ as follows:


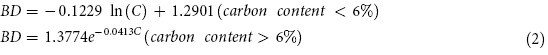


A linear regression equation between the C:N, C:P, and N:P ratios and the restoration age for each age group is constructed to facilitate the comparisons:


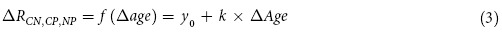


The first derivative represents the changes in the C:N, C:P, and N:P ratios in the curve; therefore, in equation 3, the first derivative of ΔR_CN,CP,NP_ versus ΔAge represents the changes in the C:N, C:P, and N:P ratios:


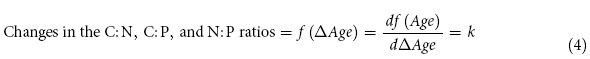


where ΔR_CN,CP,NP_ is the change in the C:N, C:P, and N:P ratios, y_0_ is a constant, k represents the slope in Equation 3, and ΔAge is the restoration age. The same method was used by Deng *et al.*^37^.

A stepwise regression analysis was used to analyze the relationship between the C:N, C:P, and N:P ratios and the annual average temperature (T, °C), the annual average precipitation (P, mm) and the restoration age (A, year) in each restoration age group. Pearson’s correlation coefficients were used to study the relationship between the soil C:N, C:P, and N:P values and the restoration age, temperature, and precipitation measured after the change in land use. Statistical analyses were performed using SPSS, ver. 17.5 (SPSS Inc., Chicago, IL, USA). The Figures were plotted using Origin 7.5 and Arcgis9.3.

## Additional Information

**How to cite this article**: Fazhu, Z. *et al.* Land use changes influences the soil C, N, and P stoichiometry under 'Grain-to-Green Program' in China. *Sci. Rep.* **5**, 10195; doi: 10.1038/srep10195 (2015).

## Figures and Tables

**Table 1:** Stepwise regression to detect factors affecting soil C:N, C:P, and N:P value at different restoration ages.

	**Restoration age**	**Equation**	**R**	**Sig.(P)**
C:N	<10	RCN = 18.10 + 2.81T–0.04P	0.06	0.01*
	10–20	RCN = 20.20–0.04P + 3.15T	0.11	0.01*
	>20	RCN = 41.06–0.05P + 2.68T	0.08	0.004**
C:P	<10	RCP = –10.45 + 0.13P–1.08T	0.29	0.000**
	10–20	RCP = –29.56 + 0.24P + 24.84T	0.45	0.000**
	>20	RCP = 9.55 + 0.21P–9.29T + 0.70A	0.33	0.000**
N:P	<10	RNP = 3.79 + 0.37A	0.34	0.000**
	10–20	RNP = 12.46 + 0.18A + 0.25T–0.02P	0.28	0.007**
	>20	RNP = 30.95 + 0.02P–0.62T + 0.08A	0.17	0.030*

Note: R_CN_, R_CP_, and R_NP_ are soil C:N, C:P, and N:P value, respectively; T (°C) is annual average temperature; P (mm) is annual average precipitation; and A (year) is restoration age after land use change.

^*^indicates Significant at P < 0.05 and.

^**^indicates extremely significant at P < 0.01.

**Table 2:** effects of annual average temperature, average precipitation, land use change, restoration age, and there interactions on soil C:N, C:P, and N:P ratios under ‘Grain-to-Green Program.

	**C:N**			**C:P**			**N:P**		
	**df**	**F**	**Sig.(P)**	**df**	**F**	**Sig.(P)**	**df**	**F**	**Sig.(P)**
T	2	0.636	0.530	2	13.363	0.000**	2	7.147	0.031*
P	2	6.662	0.030*	2	14.450	0.000**	2	10.814	0.000**
L	2	11.236	0.000**	2	4.400	0.013*	2	8.217	0.000**
A	2	10.245	0.000**	2	8.171	0.000**	2	13.780	0.000**
T × P	2	0.847	0.429	2	5.306	0.005**	3	0.972	0.406
T × L	4	0.491	0.742	4	0.867	0.484	4	0.305	0.875
T × A	4	0.634	0.638	4	1.393	0.235	4	2.000	0.093
P × L	4	4.382	0.033*	4	0.510	0.728	4	0.522	0.720
P × A	4	0.255	0.906	4	1.410	0.229	4	1.985	0.096
L × A	4	0.078	0.989	4	2.499	0.042	4	0.463	0.763
T × P × L	3	0.420	0.739	2	0.346	0.707	2	0.205	0.815
T × L × A	5	0.684	0.636	4	0.377	0.825	4	0.480	0.751
P × L × A	5	0.448	0.815	4	1.058	0.377	4	0.340	0.851
T × P × L × A	3	0.606	0.611	3	4.174	0.006**	2	1.099	0.334

Note: T, P, L, and A are the annual average temperature, average precipitation, land use change, restoration age, respectively.

^*^indicates Significant at P < 0.05 and.

^**^indicates extremely significant at P < 0.01.

**Table 3:** Correlations among soil organic C (g/kg), total N (g/kg) and total P (g/kg) under ‘Grain-to-Green Program’.

**Independent variables**	**Dependent variables**	**Number of samples**	**Correlation coefficient (R)**	**P**
Soil C	Soil N	557	0.71	<0.0001
Soil C	Soil P	394	0.28	<0.0001
Soil N	Soil P	394	0.48	<0.0001

**Table 4:** soils C, N and P stoichiometry for other countries under different land use types.

			**Mean**			
	**Soil from**	**Number soils**	**C:N**	**C:P**	**N:P**	**Reference**
Forest	New Zealand	12	20.58	358.27	16.97	Ross *et al.* (1999)
	India	19	11.59	237.92	30.91	Barbhuiya *et al.* (2004);
						Deve and Yadava (2006);
						Singh and Singh (1995);
						Srivastava (1998)
	Costa Rica	4	12.93	234.04	18.23	Cleveland *et al.* (2004)
	Germany	3	18.26	34.52	1.91	Joergensen *et al.* (1996)
	UK	2	10.37	103.36	9.97	Turner *et al.* (2001)
	Slovak	2	16.39	227.04	13.63	Kopacek *et al.* (2004)
	USA	10	25.50	90.00	3.50	Liptzin *et al.* (2013)
	Average		16.52	183.59	13.59	
	China		14.51	144.90	3.93	This study
Shurbland	Australia	5	20.5	289.00	14.25	Bui *et al.* (2013)
	China		15.00	75.16	4.8	This study
Grassland	Australia	10	18.00	173.50	9.30	Bui *et al.* (2013)
	New Zealand	22	16.60	69.50	4.00	Mulder *et al.* (2009)
	India	6	9.95	206.12	20.71	Srivastava *et al.* (1988)
	UK	29	13.82	351.40	25.19	Turner *et al.* (2001)
	Panama	4	17.11	459.82	26.87	Yavitt *et al.* (1992)
	Australia	2	14.00	54.50	3.50	Bui *et al.* (2013)
	USA	72	13.80	166.00	12.30	Cleveland *et al* (2007)
	Poland	15	17.36	308.06	17.74	Kopáček *et al.* (2004)
	Average		14.66	230.77	15.76	
	China		12.52	58.22	9.42	This study

## References

[b1] Elser J. J. & Urabe J. The stoichiometry of consumer-driven nutrient recycling: theory, observations, and consequences. Ecology, 80, 735–751 (1999).

[b2] Zhang H. L., Li J. Y., Ying Q., Guven B. B. & Olaguer E. Source Apportionment of Formaldehyde during TexAQS 2006 using a Source-Oriented Chemical Transport Model. J. Geophys. Res., 118, 1525–1535 (2013).

[b3] Michaels A. F. The ratios of life. Science, 300, 906–907 (2003).

[b4] Redfield A. C. The biological control of chemical factors in the environment. AM SCI, 46, 205–211 (1958). 24545739

[b5] Manzoni S., Trofymow J. A., Jackson R. B. & Porporato A. Stoichiometric controls on carbon, nitrogen, and phosphorus dynamics in decomposing litter. Ecology Monographys, 80, 89–106 (2010).

[b6] Zhong S. Z., Xiao L. S., Xian G. L. & Zhen S. X. Ecological stoichiometry of carbon, nitrogen, and phosphorus in estuarine wetland soils: influences of vegetation coverage, plant communities, geomorphology, and seawalls. J. Soil Sediment, 13, 1043–1051 (2013).

[b7] Li Y. J. *et al.* Is the C:N:P stoichiometry in soil and soil microbial biomass related to the landscape and land use in southern subtropical China? Global Biogeochem. CY, 26, 1–14 (2012).

[b8] Zhang Z. S., Song X. L., Lu X. G., Xue Z. S. Ecological stoichiometry of carbon, nitrogen, and phosphorus in estuarine wetland soils: influences of vegetation coverage, plant communities, geomorphology, and seawalls. J Soil Sediment, 13, 1043–1051 (2013).

[b9] Han W. X., Fang J. Y., Guo D. L. & Zhang Y. Leaf nitrogen and phosphorus stoichiometry across 753 terrestrial plant species in China. New Phytol., 168, 377–385 (2005). 16219077 10.1111/j.1469-8137.2005.01530.x

[b10] McGroddy M. E., Daufresne T. & Hedin L. O. Scaling of C:N :P stoichiometry in forests worldwide: implications of terrestrial Redfield-type ratios. Ecology, 85, 2390–2401 (2004).

[b11] Liu Z. F., Fu B. J., Zheng X. X. & Liu G. H. Plant biomass, soil water content and soil N:P ratio regulating soil microbial functional diversity in a temperate steppe : A regional scale study. Soil Biol. Biochem., 42, 445–450 (2010).

[b12] Cleveland C. C. & Liptzin D. C:N:P stoichiometry in soil: is there a “Redfield ratio” for the microbial biomass? Biogeochemistry, 85, 235–252 (2007).

[b13] Walker T. W. & Adams A. F. R. Studies on soil organic matter. Soil Sci, 85, 307–318 (1958).

[b14] Melillo J. M., Field C. B. & Moldan B. Interactions of the major biogeochemical cycles: global change and human impacts. Scientific committee on problems of the environment (SCOPE) series, vol 61. Island Press, Washington, USA (2003).

[b15] Vitousek P. M. Nutrient cycling and limitation: Hawai’I as a model system. Princeton University Press, Princeton, 2004).

[b16] Paul E. A. (ed). Soil microbiology, ecology, and biochemistry, 3rd edn. Academic press, London, 2006).

[b17] Bui E. N. & Henderson B. L. C:N:P stoichiometry in Australian soils with respect to vegetation and environmental factors. Plant. Soil., 373, 553–568 (2013).

[b18] Peñuelas J., Sardans J., Rivas-ubach A. & Janssens I. A. The human induced imbalance between C, N and P in Earth’s life system. Global Change. Biol., 18, 3–6 (2012).

[b19] Tian H. Q., Chen G. S., Zhang C., Melillo J. M. & Hall C. A. S. Pattern and variation of C:N:P ratios in China’s soils: a synthesis of observational data. Biogeochemistry, 98, 139–151 (2010).

[b20] Lal R. Promise and limitations of soils to minimize climate change. J SOIL WATER CONSERV, 63, 113–118 (2008).

[b21] Cristina A., Teodoro M. & Luis V. G. Microbial C, N and P in soils of Mediterranean oak forests: influence of season, canopy cover and soil depth. Biogeochemistry, 101, 77–92 (2010).

[b22] Lü Y. H. *et al.* A Policy-Driven Large Scale Ecological Restoration: Quantifying Ecosystem Services Changes in the Loess Plateau of China. PLos One, 7: e31782. 10.1371/journal.pone.0031782 (2012). 22359628 PMC3280995

[b23] Fu B. J., Chen L. D., Qiu Y., Wang J. & Meng Q. H. Land Use Structure and Ecological Processes in the Losses Hilly Area. Chinese Commercial Press, Beijing, 2002).

[b24] Uchida E., Rozelle S. & Xu J. Conservation payments, liquidity constraints, and off-farm labor: impact of the Grain–for–Green Program on rural households in China. Am J Agr. Econ., 91, 70–86 (2009).

[b25] Zhao F. Z., Han X. H., Yang G. H., Feng Y. Z., Ren G. X. Soil structure and carbon distribution in subsoil affected by vegetation restoration. Plant Soil Environ, 60, 21–26 (2014).

[b26] Chang R. Y., Fu B. J., Liu G. H. & Liu S. G. Soil Carbon Sequestration Potential for “Grain for Green” Project in Loess Plateau, China. Environ Manage, 48, 1158–1172 (2011). 21553107 10.1007/s00267-011-9682-8

[b27] Bui E. N. & Henderson E. N. C:N:P stoichiometry in Australian soils with respect to vegetation and environmental factors. Plant Soil., 373, 553–568 (2013).

[b28] Zeng D. H. & Chen G. S. Ecological stoichiometry: a science to explore the complexity of living systems. Acta Phyto. ecologica Sin. 29, 1007–1019 (2005).

[b29] He J. S. & Han X. G. Ecological stoichiometry: searching for unifying principles from individuals to ecosystems. J Plant. Eco., 31, 2–6 (2010).

[b30] Aponte C., Marañón, T., García, L. V. Microbial C, N and P in soils of Mediterranean oak forests: Influence of season, canopy cover and soil depth, Biogeochemistry, 101, 77–92 (2010).

[b31] Zhang H. *et al.* Sampling Date, Leaf Age and Root Siz e: Implicatio ns for the Study of Plant C:N:P Stoichiom etry. PLos One 8: e603 60. doi:10.1 371/journal.pone. 0060360 (2013). 10.1371/journal.pone.0060360 PMC3614965 23565234

[b32] Oleksyn J., Reich P. B., Zytkowiak R., Karolewski P. & Tjoelker M. G. Nutrient conservation increases with latitude of origin in EuropeanPinus sylvestris populations. Oecologia, 136, 220–235 (2003). 12756524 10.1007/s00442-003-1265-9

[b33] Yang Y. H., Luo Y. Q. & Finzi A. C. Carbon and nitrogen dynamics during forest stand development: a global synthesis. New Phytol., 190, 977–989 (2011). 21323927 10.1111/j.1469-8137.2011.03645.x

[b34] Shi S. W., Zhang W., Zhang P., Yu Y. Q. & Ding F. A synthesis of change in deep soil organic carbon stores with afforestation of agricultural soils. Forest Ecol. Manag., 296, 53–63 (2013).

[b35] Zhao F. Z. *et al.* Policy-Guided Nationwide Ecological Recovery: Soil Carbon Sequestration Changes Associated With the Grain-to-Green Program in China. Soil. sci., 10, 550–555 (2013).

[b36] Kirkby C. A. *et al.* Stable soil organic matter: a comparison of C: N: P: S ratios in Australian and other world soils. Geoderma, 163, 197–208 (2011).

[b37] Deng L., Liu G. B. & Shangguan Z. P. Land use conversion and changing soil carbon stocks in China’s ‘Grain-for-Green’ Program’: a synthesis. Global Change Biol., 20, 3544–3556 (2014). 10.1111/gcb.12508 24357470

[b38] Guo L. B. & Gifford R. M. Soil carbon stocks and land use change: a Meta analysis. Global Change Biol., 8, 345–360 (2002).

